# X-linked inhibitor of apoptosis protein (XIAP) predicts disease-free survival in *BRAFV600E* mutant papillary thyroid carcinoma in middle eastern patients

**DOI:** 10.3389/fendo.2022.1054882

**Published:** 2022-12-12

**Authors:** Sandeep Kumar Parvathareddy, Abdul K. Siraj, Rong Bu, Kaleem Iqbal, Maha Al-Rasheed, Wael Al-Haqawi, Padmanaban Annaiyappanaidu, Nabil Siraj, Saeeda O. Ahmed, Saif S. Al-Sobhi, Fouad Al-Dayel, Khawla S. Al-Kuraya

**Affiliations:** ^1^ Human Cancer Genomic Research, Research Center, King Faisal Specialist Hospital and Research Centre, Riyadh, Saudi Arabia; ^2^ Department of Surgery, King Faisal Specialist Hospital and Research Centre, Riyadh, Saudi Arabia; ^3^ Department of Pathology, King Faisal Specialist Hospital and Research Centre, Riyadh, Saudi Arabia

**Keywords:** papillary thyroid carcinoma, XIAP, BRAFV600E, disease-free survival, prognosis

## Abstract

**Background:**

X-linked inhibitor of apoptosis (XIAP) is the most potent caspase inhibitory IAP family member and its over-expression is implicated in aggressive behavior of various solid tumors, including papillary thyroid carcinoma (PTC). *BRAFV600E* mutation is the most common oncogenic event in PTC and is also known to be associated with aggressive clinico-pathological characteristics. In this study, we investigated the prevalence of XIAP expression in more than 1600 PTCs from Middle Eastern ethnicity and its prognostic value to predict disease-free survival (DFS), in combination with the *BRAFV600E* mutation.

**Methods:**

Clinical data, XIAP expression by immunohistochemistry and *BRAF* mutation status were analyzed in 1640 Saudi PTC patients seen at our institute between 1988 - 2020.

**Results:**

*BRAFV600E* mutation was found in 910 of 1640 patients (55.5%) and was significantly correlated with older age, extrathyroidal extension, bilaterality, multifocality and lymph node metastasis, but was not an independent predictor of DFS. XIAP was over-expressed in 758 of 1640 (46.2%) and was associated with aggressive clinico-pathological features. It was also found to be an independent prognostic marker for DFS (HR = 1.28, 95% CI = 1.02 – 1.60, P = 0.0342). XIAP overexpression was correlated with presence of *BRAFV600E* mutation in PTC patients. Interestingly, we found the ability to predict shorter DFS was 2.7-fold higher in PTCs with over-expression of XIAP and *BRAFV600E* mutation compared to patients with high XIAP and wild-type *BRAFV600E* status (HR = 2.74, 95% CI = 2.19 – 3.44, p < 0.0001).

**Conclusion:**

XIAP expression is an independent predictor of prognosis in Middle Eastern PTC patients. Combination of XIAP expression and *BRAFV600E* mutation can synergistically improve the DFS prediction in PTC patients, which may help clinicians to establish the most appropriate initial care and long-term surveillance strategies.

## Introduction

Thyroid carcinoma is the most common endocrine malignancy (1) and papillary thyroid carcinoma (PTC) accounts for the majority (~90%) of all thyroid cancers (2, 3). In Saudi Arabia, PTC is the second commonest cancer affecting women, after breast cancer (4). Despite the indolent course and excellent prognosis of PTC, there are some patients who present with aggressive disease and poor prognosis, which remains a major concern for clinicians (5, 6). Identifying molecular markers that can predict poor prognosis is of paramount importance in helping healthcare providers to tailor therapy and follow-up of PTC patients.

The role of genetic alteration in MAPK signaling pathway in PTC initiation and progression has been previously well documented (7, 8). A *BRAFV600E* point mutation in exon 15 is commonly detected in PTC and accounts for more than 90% of all *BRAF* mutations (9). *BRAF* mutation is present in more than 50% of PTCs (10, 11). Numerous studies have documented the oncogenic molecular mechanisms of *BRAFV600E* in driving aggressiveness of PTC (11–14). Despite the strong association between *BRAF* mutation and aggressive clinico-pathological features, its ability as a sole marker to predict prognosis is still a subject of debate. While some studies have clearly demonstrated that *BRAFV600E* significantly affected prognosis (12, 15–19), others have failed to establish *BRAF* mutation as an independent prognostic factor in PTC patients (14, 20–22). Therefore, synergistic interaction between *BRAF* mutations and other oncogenic markers may better predict prognosis of PTC.

The known marker that has been confirmed by us previously to play an important functional and prognostic role in PTC is the X-linked inhibitor of apoptosis protein (XIAP) (23). The inhibitor of apoptosis is a family of endogenous caspase inhibitors that share a common Baculoviral IAP repeat domain (24, 25). XIAP is the best characterized member of the IAP family in terms of the caspase inhibitory mechanism (26). XIAP dysregulation has been shown to be associated with aggressive clinical behavior and worse outcome in several cancers, including PTC (27–31).

Therefore, we hypothesized that synergistic association between *BRAFV600E* mutation and XIAP alteration may better predict the prognosis of PTC. The aim of the present study was to investigate whether *BRAFV600E* mutational status is associated with XIAP alteration and whether these associations can synergistically improve the ability to predict prognosis in a large cohort of Middle Eastern PTC patients.

## Materials and methods

### Patient selection and clinico-pathological data

One thousand seven-hundred and sixteen consecutive unselected PTC patients diagnosed between 1988 and 2020 at King Faisal Specialist Hospital and Research Centre (Riyadh, Saudi Arabia) were available to be included in the study. However, complete data for XIAP expression and *BRAF* mutation were available for 1640 PTC patients and hence only these cases were included for further analysis. Cases were identified based on clinical history followed by fine needle aspiration cytology for confirmation. The Institutional Review Board of the hospital approved this study. Since only retrospective patient data was utilized, the Research Advisory Council (RAC) provided waiver of consent under project RAC # 221 1168 and # 2110 031.

Baseline clinico-pathological data were collected from case records and have been summarized in **Table 1**. Staging of PTC was performed using the eighth edition of American Joint Committee on Cancer (AJCC) staging system (32).

**Table 1:** Clinico-pathologic characteristics of papillary thyroid carcinoma.

	Total	
	No.	%
**No. of patients**	1640	
**Age (Yrs)**		
Median (range)	38.2 (5.9 – 88.0)	
≤ 55	1337	81.5
> 55	303	18.5
**Sex**		
Female	1238	75.5
Male	402	24.5
**Histology Type**		
Classical Variant	1025	62.5
Follicular Variant	287	17.5
Tall-Cell Variant	178	10.8
Other variants	150	9.2
**Tumor laterality**		
Unilateral	1102	67.8
Bilateral	538	32.8
**Tumor focality**		
Unifocal	805	49.1
Multifocal	835	50.9
**Extrathyroidal extension**		
Absent	957	58.3
Present	683	41.7
**Lymphovascular invasion**		
Absent	1180	71.9
Present	460	28.1
**pT**		
T1	652	39.7
T2	534	32.6
T3	331	20.2
T4	120	7.3
Unknown	3	0.2
**pN**		
pN0	683	41.6
pN1	804	49.0
pNx	153	9.3
**pM**		
pM0	1565	95.4
pM1	75	4.6
**Stage**		
I	1385	84.4
II	177	10.8
III	24	1.5
IV	48	2.9
Unknown	6	0.4

### *BRAFV600E* mutation analysis

*BRAFV600E* mutation data was assessed in our laboratory by Sanger sequencing and has been published by us previously (33).

### Tissue microarray construction and immunohistochemistry analysis

Tissue microarray (TMA) format was utilized for immunohistochemical analysis of the PTC samples. TMA was constructed as previously described (34). Briefly, modified semiautomatic robotic precision instrument (Beecher Instruments, Woodland, WI) was used to punch tissue cylinders with a diameter of 0.6 mm from representative tumor area of the donor tissue block and brought into the recipient paraffin block. Two 0.6-mm cores of PTC were arrayed from each case.

XIAP immunohistochemical staining was performed on 1022 PTC samples previously by us (23). We expanded the staining to 1640 cases using the same protocol. Scoring (H-score) and cutoff for analysis was also performed as described previously (23). Briefly, X-tile plots were constructed for assessment of biomarker and optimization of cutoff points based on outcome as has been described earlier (35). PTCs were categorised into two groups based on X-tile plots: one with complete absence or reduced staining of XIAP (H score = 0–40) and the other group showed overexpression of XIAP (H score > 40).

### Follow-up and study endpoint

Patients were regularly followed by both physical examinations and imaging studies to identify tumor recurrence. The median follow-up was 7.5 years (range 1.0 – 30.2 years). The study end-point was disease-free survival (DFS). Patients were grouped according to disease status, with patients considered to be disease-free in the absence of clinical, biochemical (unstimulated serum thyroglobulin (Tg) levels of < 0.2 µg/L or stimulated Tg levels of < 1 µg/L in the absence of interfering thyroglobulin antibodies (TgAb)) or radiological evidence of disease persistence or recurrence. In contrast, active disease was defined by the presence of unstimulated serum Tg levels ≥ 0.2 µg/L or stimulated Tg levels ≥1 µg/L; a rising or denovo appearance of TgAb; or abnormal findings on radio-imaging.

### Statistical analysis

The associations between clinico-pathological variables and XIAP expression/*BRAFV600E* mutation was performed using contingency table analysis and Chi square tests. Mantel-Cox log-rank test was used to evaluate DFS. Survival curves were generated using the Kaplan-Meier method. Cox proportional hazards model was used for univariate and multivariate analysis. Two-sided tests were used for statistical analyses with a limit of significance defined as p value < 0.05. Data analyses were performed using the JMP14.0 (SAS Institute, Inc., Cary, NC) software package.

## Results

### Patient and tumor characteristics

Median age of the study population was 38.2 years (range: 5.9 – 88 years), with a male to female ratio of 1:3. The majority of tumors were classical variant of PTC (62.5%; 1025/1640). 32.8% (538/1640) of tumors were bilateral and 50.9% (835/1640) were multifocal. 41.7% (683/1640) of tumors exhibited extrathyroidal extension and 28.1% (460/1640) showed lymphovascular invasion. Lymph node metastasis was noted in 49.0% (804/1640) and distant metastasis in 4.6% (75/1640) of PTCs (**Table 1**).

### Frequency of *BRAFV600E* mutation and its clinico-pathological associations

*BRAFV600E* mutation was noted in 55.5% (910/1640) of PTCs in our cohort and was significantly associated with adverse clinico-pathological characteristics such as older age at diagnosis (p < 0.0001), tall cell variant of PTC (p < 0.0001), bilateral tumors (p = 0.0001), multifocality (p = 0.0004), extrathyroidal extension (p < 0.0001) and lymph node metastasis (p < 0.0001). Interestingly, we also found a significant association between *BRAFV600E* mutation and XIAP over-expression (p < 0.0001) (**Table 2**).

**Table 2:** Clinico-pathologic associations of *BRAFV600E* mutation in papillary thyroid carcinoma.

	Total		*BRAF* wildtype		*BRAFV600E* mutant		p value
	No.	%	No.	%	No.	%	
**No. of patients**	1640		730	44.5	910	55.5	
**Age (Yrs)**							
≤ 55	1337	81.5	631	86.4	706	77.6	< 0.0001
> 55	303	18.5	99	13.6	204	22.4	
**Sex**							
Female	1238	75.5	566	77.5	672	73.8	0.0837
Male	402	24.5	164	22.5	238	26.2	
**Histology Type**							
Classical Variant	1025	62.5	380	52.1	645	70.9	< 0.0001
Follicular Variant	287	17.5	222	30.4	65	7.1	
Tall-Cell Variant	178	10.8	27	3.7	151	16.6	
Other variants	150	9.2	101	13.8	49	5.4	
**Tumor laterality**							
Unilateral	1102	67.8	527	72.2	575	63.2	0.0001
Bilateral	538	32.8	203	27.8	335	36.8	
**Tumor focality**							
Unifocal	805	49.1	394	54.0	411	45.2	0.0004
Multifocal	835	50.9	336	46.0	499	54.8	
**Extrathyroidal extension**							
Absent	957	58.3	517	70.8	440	48.4	< 0.0001
Present	683	41.7	213	29.2	470	51.6	
**Lymphovascular invasion**							
Absent	1180	71.9	526	72.0	654	71.9	0.9333
Present	460	28.1	204	28.0	256	28.1	
**pT**							
T1	652	39.8	274	37.6	378	41.6	0.0007
T2	534	32.6	242	33.2	292	32.1	
T3	331	20.2	173	23.8	158	17.4	
T4	120	7.3	39	5.4	81	8.9	
**pN**							
pN0	683	45.9	365	55.5	318	38.4	< 0.0001
pN1	804	54.1	293	44.5	511	61.6	
**pM**							
pM0	1565	95.4	687	94.1	878	96.5	0.0227
pM1	75	4.6	43	5.9	32	3.5	
**Stage**							
I	1385	84.8	631	86.8	754	83.1	0.1006
II	177	10.8	70	9.6	107	11.8	
III	24	1.5	6	0.8	18	2.0	
IV	48	2.9	20	2.8	28	3.1	
**XIAP IHC**							
Low	882	53.8	506	69.3	376	41.3	< 0.0001
High	758	46.2	224	30.7	534	58.7	

### XIAP expression and its clinico-pathological associations

XIAP over-expression was seen in 46.2% (758/1640) of PTCs. Over-expression of XIAP was found to be associated with older age at diagnosis (p < 0.0001), tall cell variant of PTC (p < 0.0001), extrathyroidal extension (p < 0.0001), larger tumor size (p = 0.0007), lymph node metastasis (p = 0.0023) and advanced stage (p < 0.0001) (**Table 3**).

**Table 3:** Clinico-pathologic associations of XIAP expression in papillary thyroid carcinoma.

	Total		XIAP Low		XIAP High		p value
	No.	%	No.	%	No.	%	
**No. of patients**	1640		882	53.8	758	46.2	
**Age (Yrs)**							
≤55	1337	81.5	751	85.1	586	77.3	< 0.0001
>55	303	18.5	131	14.9	172	22.7	
**Sex**							
Female	1238	75.5	675	76.5	563	74.3	0.2899
Male	402	24.5	207	23.5	195	25.7	
**Histology Type**							
Classical Variant	1025	62.5	520	59.0	505	66.6	< 0.0001
Follicular Variant	287	17.5	210	23.8	77	10.2	
Tall-Cell Variant	178	10.8	61	6.9	117	15.4	
Other variants	150	9.2	91	10.3	59	7.8	
**Tumor laterality**							
Unilateral	1102	67.8	604	68.5	498	65.7	0.2318
Bilateral	538	32.8	278	31.5	260	34.3	
**Tumor focality**							
Unifocal	805	49.1	440	49.9	365	48.2	0.4838
Multifocal	835	50.9	442	50.1	393	51.8	
**Extrathyroidal extension**							
Absent	957	58.3	579	65.6	378	49.9	< 0.0001
Present	683	41.7	303	34.4	380	50.1	
**Lymphovascular invasion**							
Absent	1180	71.9	652	73.9	528	69.7	0.0554
Present	460	28.1	230	26.1	230	30.3	
**pT**							
T1	652	39.8	365	41.5	287	37.9	0.0007
T2	534	32.6	289	32.9	245	32.3	
T3	331	20.2	182	20.7	149	19.7	
T4	120	7.3	43	4.9	77	10.2	
**pN**							
pN0	683	45.9	393	49.6	290	41.7	0.0023
pN1	804	54.1	399	50.4	405	58.3	
**pM**							
pM0	1565	95.4	847	96.0	718	94.7	0.2067
pM1	75	4.6	35	4.0	40	5.3	
**Stage**							
I	1385	84.8	776	88.3	609	80.7	< 0.0001
II	177	10.8	80	9.1	97	12.8	
III	24	1.5	11	1.2	13	1.7	
IV	48	2.9	12	1.4	36	4.8	

### Prediction of disease-free survival by XIAP expression and *BRAFV600E* mutation status

We next analyzed the ability of XIAP and *BRAFV600E* to predict outcome in PTC patients in our cohort. Both XIAP and *BRAFV600E* could predict DFS on univariate analysis (XIAP: Hazard ratio (HR) = 1.74, 95% confidence interval (CI) = 1.41 – 2.15, p value < 0.0001; *BRAFV600E*: HR = 1.64, 95% CI = 1.32 – 2.05, P < 0.0001) (**Table 4**). However, on multivariate analysis (after adjusting for age, sex, laterality, tumor size, focality, extrathyroidal extension, lymph node metastasis and distant metastasis), XIAP was found to be an independent predictor of DFS (HR = 1.28, 95% CI = 1.02 – 1.60, P = 0.0342) but *BRAFV600E* was not (HR = 1.22, 95% CI = 0.96 – 1.56, P = 0.1041) (**Table 4**).

**Table 4:** Comparison of multivariate analysis to predict DFS using the XIAP status alone or in combination with *BRAFV600E* mutation.

Groups	n	Events	Unadjusted HR (95% CI)	P value	Adjusted* HR (95% CI)	P value
XIAP low	882	123	Reference		Reference	
XIAP high	758	232	1.74 (1.41 – 2.15)	< 0.0001	1.28 (1.02 – 1.60)	0.0342
*BRAFV600E* wildtype	730	119	Reference		Reference	
*BRAFV600E* mutant	910	236	1.64 (1.32 – 2.05)	< 0.0001	1.22 (0.96 – 1.56)	0.1041
XIAP low	882	123	Reference		Reference	
XIAP(+) *BRAFV600E*(+)	545	194	1.87 (1.47 – 2.40)	< 0.0001	2.74 (2.19 – 3.44)	< 0.0001
XIAP(+) *BRAFV600E*(-)	213	38	1.34 (0.92 – 1.92)	0.1180	1.30 (0.88 – 1.87)	0.1787

*Adjusted for age, sex, laterality, tumor size, focality, extrathyroidal extension, lymph node metastasis and distant metastasis.

HR, Hazard ratio; CI, Confidence Interval.

**Figure 1:**
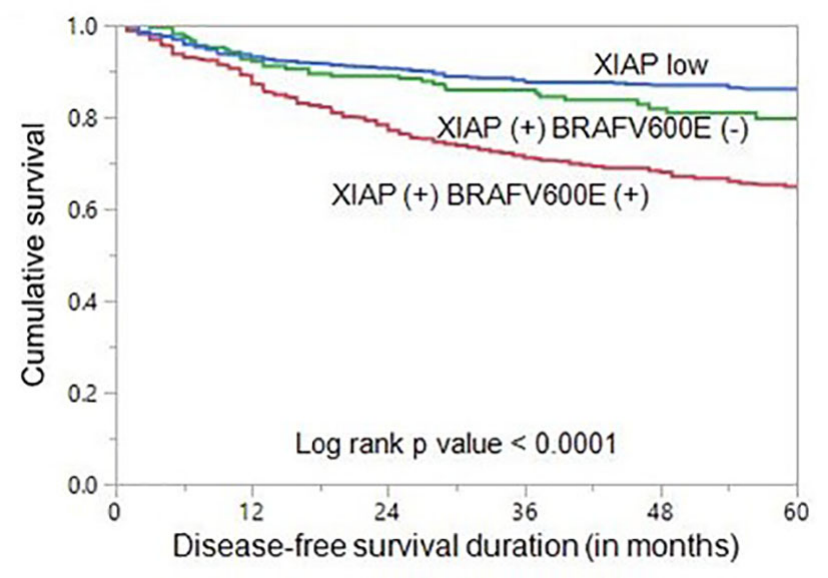
Disease-free survival (DFS) in papillary thyroid carcinoma (PTC). Kaplan-Meier survival curve showing shorter DFS in patients with co-existing XIAP over-expression and BRAF mutation compared to those with co-existing XIAP over-expression and BRAF wildtype or XIAP low expression groups (p < 0.0001).

To determine whether the combination of XIAP and *BRAFV600E* could be a better predictor of prognosis in our cohort, we classified the patients into three groups: XIAP low expression, XIAP over-expression and *BRAFV600E* mutant, XIAP over-expression and *BRAFV600E* wildtype. Indeed, the combination of XIAP over-expression and *BRAFV600E* mutation was found to be a better independent predictor of DFS (HR = 2.74, 95% CI = 2.19 – 3.44, p < 0.0001) in our cohort (**Table 4**; **Figure 1**).

## Discussion

Our study offers further evidence that the *BRAFV600E* mutation is associated with aggressive form of PTC in Middle Eastern patients. The *BRAFV600E* mutation is the most common mutation in PTC and occurs in 40-80% of PTCs (36). The prevalence of *BRAFV600E* in this cohort was 55.5%. Despite the association with aggressive clinico-pathological characteristics in our cohort such as older age, bilateral tumors, multifocality, extrathyroidal extension and lymphnode metastasis, *BRAFV600E* mutation alone was not found to be an independent predictor of disease-free survival in Middle Eastern patients with PTC. Previous reports have been controversial regarding the prognostic significance of *BRAFV600E* mutation, with some studies identifying *BRAFV600E* mutation as an independent prognostic marker in PTC (15–19), whereas other studies have failed to show the prognostic value of *BRAFV600E* mutation (14, 20–22). The prevalence of *BRAFV600E* mutation, cohort size, patients’ selection criteria and ethnicity might contribute to these differences.

Given the high survival among thyroid cancer, there is a need to identify molecular marker to predict prognosis for continued surveillance and help in clinical decision making for PTC patients. We have looked for association of other markers that could improve the limited prognostic value of *BRAFV600E* mutation. In this study, we investigated whether *BRAFV600E* mutation may better predict PTC recurrence in the presence of XIAP alteration. In a previous study (23), we found XIAP to be an oncogenic marker in PTC cells. We also showed that XIAP expression was high in PTC clinical samples and was associated with aggressive clinico-pathological markers.

In this current study, we found XIAP overexpression in 46.2% of PTC cases and it was associated with older age, advanced tumor stage and lymph node metastasis among other clinico-pathological parameters. Interestingly, XIAP overexpression was strongly associated with *BRAFV600E* mutations in PTC patients. More importantly, XIAP was an independent molecular marker for shorter disease-free survival in multivariate analysis. The ability of XIAP to predict prognosis in PTC patient and its strong association with *BRAFV600E* mutation led us to hypothesize that *BRAFV600E*, if used in combination with XIAP, may improve the ability for prediction of prognosis in PTC patients. Interestingly, upon classifying patients based on XIAP expression in tumors, we found that the ability to predict shorter disease-free survival was 2.7-fold higher in PTCs with high expression of XIAP and *BRAFV600E* mutation compared to patients with high XIAP and wild-type *BRAFV600E* status. Therefore, XIAP could be a potential prognostic biomarker that may be used in combination with *BRAFV600E* mutations in the clinical setting to predict prognosis in Middle Eastern PTC. Previous studies have demonstrated that XIAP is a NF-kappaB (NFκB)-dependent member of the IAP gene family (37, 38). MAPK signalling can also lead to the activation of the transcription factor NFκB, causing upregulation of survival genes such as XIAP (39, 40). This could be a possible explanation for the association between XIAP and *BRAF*, as well as their ability to better predict prognosis in PTC when used in combination.

A previous study has reported the association between *BRAFV600E* mutation and molecular marker XIAP, in predicting patients’ outcome (41). They conducted a study on 164 PTC patients from South Korea and found that PTCs positive for *BRAFV600E* mutation and negative for XIAP expression had significantly higher rate of recurrence. This is inconsistent with our study, where we found that *BRAFV600E* mutations in XIAP positive PTC was more useful in improving prediction of disease-free survival. Whether differences in cohort size and ethnicity played a role in this contradictory results are not clear. In addition, the younger median age of our study cohort could also be a contributing factor to the differences in results of our study compared to other studies. The younger age of our cohort most likely represents the inherent aggressive nature of PTC in the Middle Eastern ethnicity, as shown by us and other studies from this region (42–46). Another study analyzed 123 PTCs and found that presence of *BRAFV600E* mutation was unrelated to XIAP expression (47). Hence, more studies are required to establish the prognostic role of *BRAFV600E* mutation and XIAP expression in PTCs.

In summary, the XIAP expression in PTC better predicts aggressive disease and prognosis based on stratification by *BRAFV600E* mutation status. The synergistic interaction between XIAP overexpression and *BRAFV600E* mutations could potentially be helpful to clinicians in establishing optimal decision making regarding PTC patients’ therapy, follow-up and surveillance.

## Data availability statement

The original contributions presented in the study are included in the article/supplementary material. Further inquiries can be directed to the corresponding author.

## Ethics statement

The studies involving human participants were reviewed and approved by King Faisal Specialist Hospital and Research Centre. Written informed consent from the participants’ legal guardian/next of kin was not required to participate in this study in accordance with the national legislation and the institutional requirements.

## Author contributions

Study concept and design: KA-K, SP, AS, RB. Executed the study: SP, AS, RB, KI, MA-R, WA-H, PA, NS, SA, SA-S, FA-D. Statistical analysis: SP. Drafting the article: KA-K, AS, SP. Critical revision of the article for important intellectual content, writing of the article, and approval of the final version: KA-K, SP, AS, RB, KI, MA-R, WA-H, PA, NS, SA, SA-S, FA-D. All authors contributed to the article and approved the submitted version.
